# Supplementary material for: Aerosol-boundary-layer-monsoon interactions amplify semi-direct effect of biomass smoke on low cloud formation in Southeast Asia
Source: Nat Commun. 2021 Nov 5;12:6416. doi: 10.1038/s41467-021-26728-4 (PMC8571318; doi:10.1038/s41467-021-26728-4)
Supplement: Supplementary file 1 — Supplementary Information [file 41467_2021_26728_MOESM1_ESM.pdf]

Supplementary Information

for

Aerosol-boundary-layer-monsoon interactions amplify semi-direct effect of biomass smoke on low cloud formation in Southeast Asia

Ke Ding, Xin Huang, Aijun Ding, Minghuai Wang, Hang Su, Veli-Matti Kerminen, Tuukka Petäjä, Zhemin Tan, Zilin Wang, Derong Zhou, Jianning Sun, Hong Liao, Huijun Wang, Ken Carslaw, Robert Wood, Paquita Zuidema, Daniel Rosenfeld, Markku Kulmala, Congbin Fu, Ulrich Pöschl, Yafang Cheng, Meinrat O. Andreae

This PDF file includes:

Supplementary Figures 1 to 15……………………………………………..….2-16

Supplementary Tables 1 to 4………………………………………………….17-19

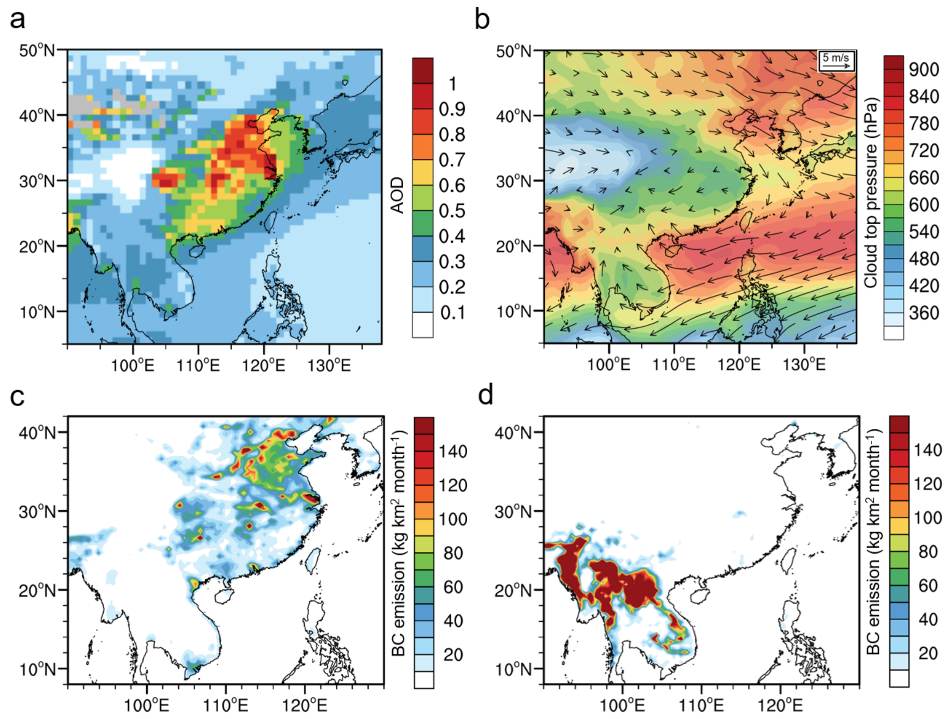

Supplementary Fig. 1 | Distribution of MODIS aerosol optical depth (AOD) and black carbon (BC) emissions from anthropogenic activities and biomass burning in East Asia. a, 2000-2015 averaged MODIS AOD for non-spring months (all months except March and April). **b**, 2000-2015 averaged cloud top pressure in March with wind field at 925 hPa. **c**, **d**, BC emission intensities from anthropogenic activities and biomass burning in March, respectively. Anthropogenic sources were derived from the MIX emission inventory, and biomass burning sources were calculated online by the 3BEM module embedded in the WRF-Chem model.

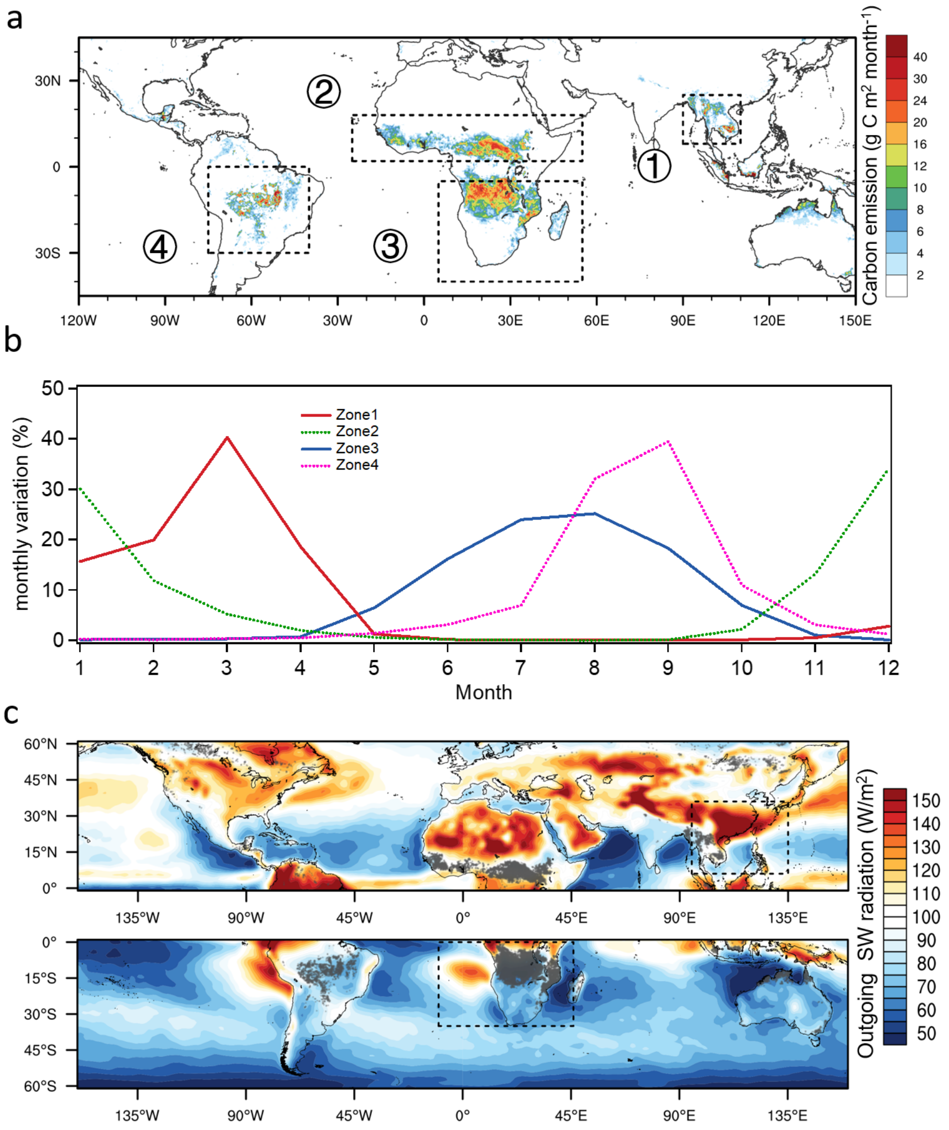

Supplementary Fig. 2 | Biomass burning (BB) carbon emission and its seasonal patterns in main BB regions in the world. a, Global distribution of annual averaged carbon emissions from biomass burning. **b,** Monthly variation of carbon emissions from four important biomass burning regions: Southeast Asia, North Africa, South Africa and Amazon. **c,** Distribution of CERES outgoing short-wave radiation and biomass burning spots in fire seasons during 2000-2015 (March for North Hemisphere and August for South Hemisphere in August). Note: Biomass burning activities are shown as grey dots based on the MODIS burned area product (MOD/MYD14A1).

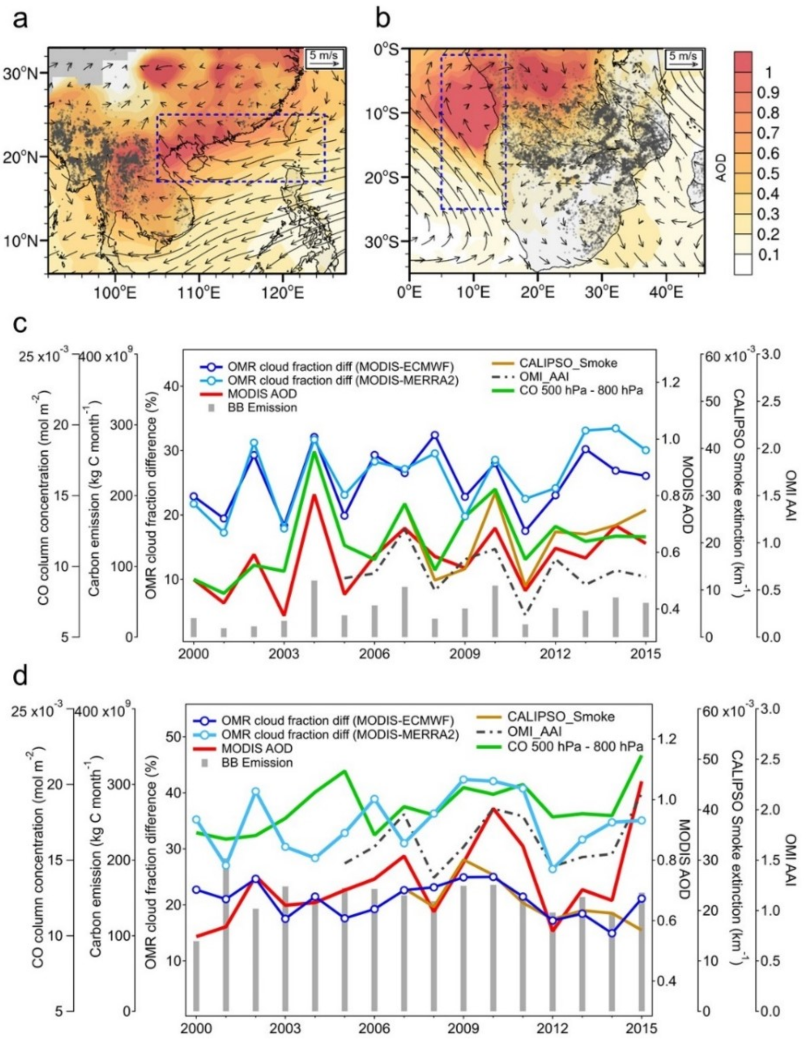

Supplementary Fig. 3 | Inter-annual variations of aerosols and low cloud enhancements in Asia and Africa. a, b, Averaged aerosol optical depth (AOD) overlaid by 925-hPa wind during 2000-2015 in March in Asia and in August in Africa. **c, d,** Time series of monthly averaged observation minus reanalysis (OMR) difference of low clouds and various aerosol indices (MODIS AOD, OMI absorption aerosol index (AAI) and CALPSO column smoke extinction) and column carbon monoxide (CO) between 500-800 hPa in the Modern-Era Retrospective analysis for Research and Applications version 2 (MERRA-2) reanalysis for Asia in March and Africa in August during 2000-2015. All data except BB emissions are averaged for low-cloud enhancement regions. The regions for averaging in (**c**) and (**d**) are defined in (**a**) and (**b**) as dashed blue boxes. Note: Correlation coefficients (R) between low cloud bias (MODIS-ECMWF and MODIS-MERRA2) and AOD are 0.77, 0.79 in Asia and 0.37, 0.54 in Africa, respectively.

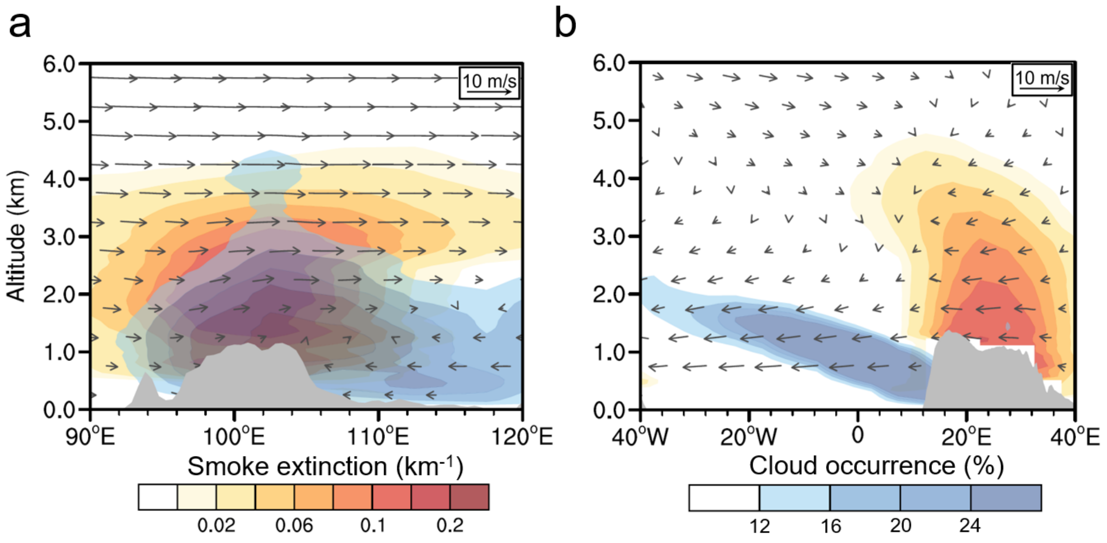

Supplementary Fig. 4 | CALIPSO vertical distribution of smoke extinction and cloud occurrence. a, Averaged vertical cross-section of smoke extinction and cloud occurrence in subtropical eastern Asia (17°N – 23°N) in March during 2007-2015. **b,** same with (**a**) but for Atlantic-Africa region (11°S – 23°S) in August.

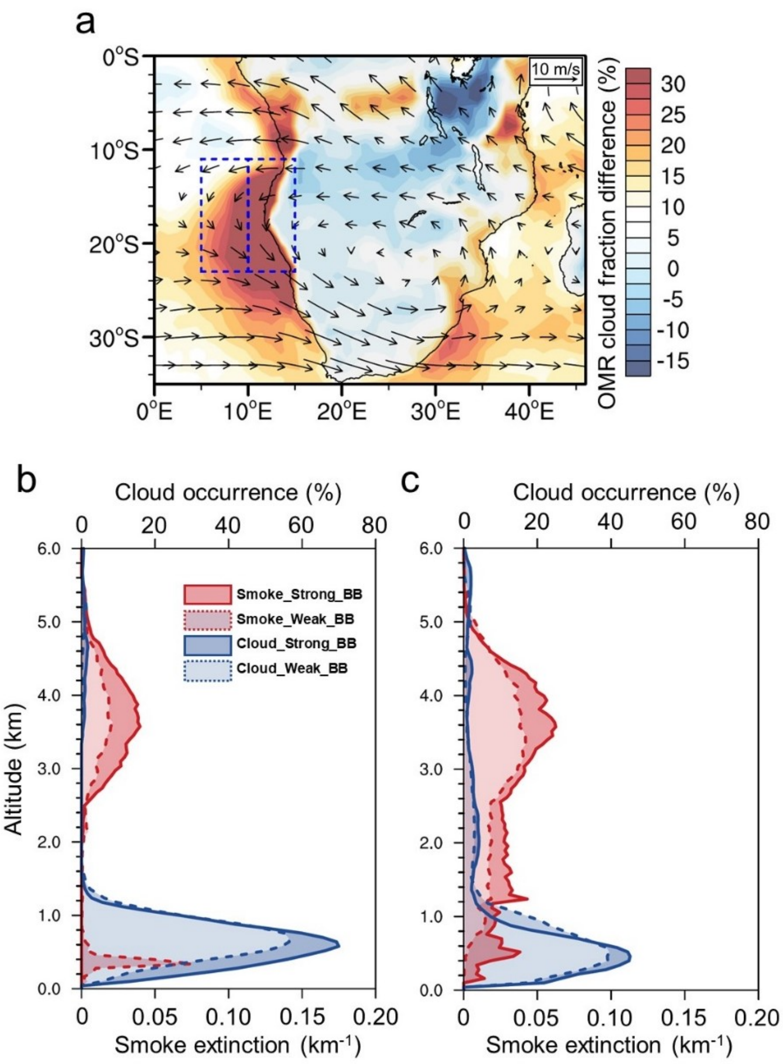

Supplementary Fig. 5 | CALIPSO vertical distribution of smoke extinction and cloud occurrence in the Africa-Atlantic region. a, Averaged observation minus reanalysis (OMR) difference in cloud fraction overlaid by 700-hPa wind during 2000-2015 in August in Africa. **b, c,** Averaged vertical profile of smoke extinction and cloud occurrence in the three highest (indicated as "Strong_BB") and lowest (indicated as "Weak_BB") upper-level smoke plume years during 2007-2015 August for **(b)** Atlantic Ocean (2007, 2008, 2009 as high years compared with 2012, 2013, 2014), and **(c)** coastal west Central Africa in the south (2010, 2011, 2014 vs. 2009, 2012, 2013), respectively. The highest and lowest three years were classified according to the column smoke extinction between 2-5 km, excluding years with inconsistent aerosol optical depth and extremely high smoke aerosols above the cloud. The regions for averaging in (**b**) and (**c**) are defined as the left and right blue boxes in (**a**).

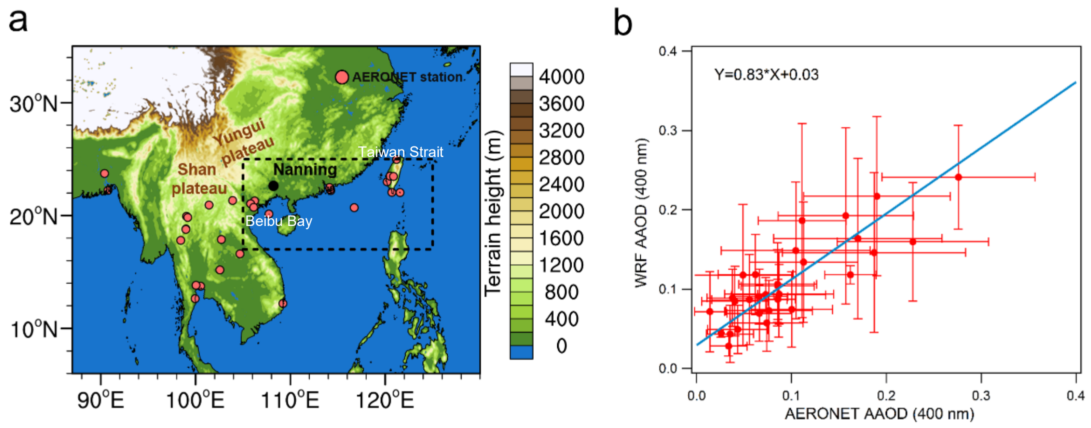

Supplementary Fig. 6 | Model validation of simulated absorptive aerosol optical depth (AAOD) along the biomass burning (BB) smoke plume. a, Locations of AERONET stations used to validate the simulated AAOD and the geographical definitions used in this study. **b,** Comparison between observed and simulated AAOD (EXP_ARI) at 400 nm wavelength at AERONET stations in March during 2001-2015. The whiskers show the standard deviation. The blue solid line gives the reduced major axis (RMA) regression for the scatters, and the fitted function is labelled in the top left corner.

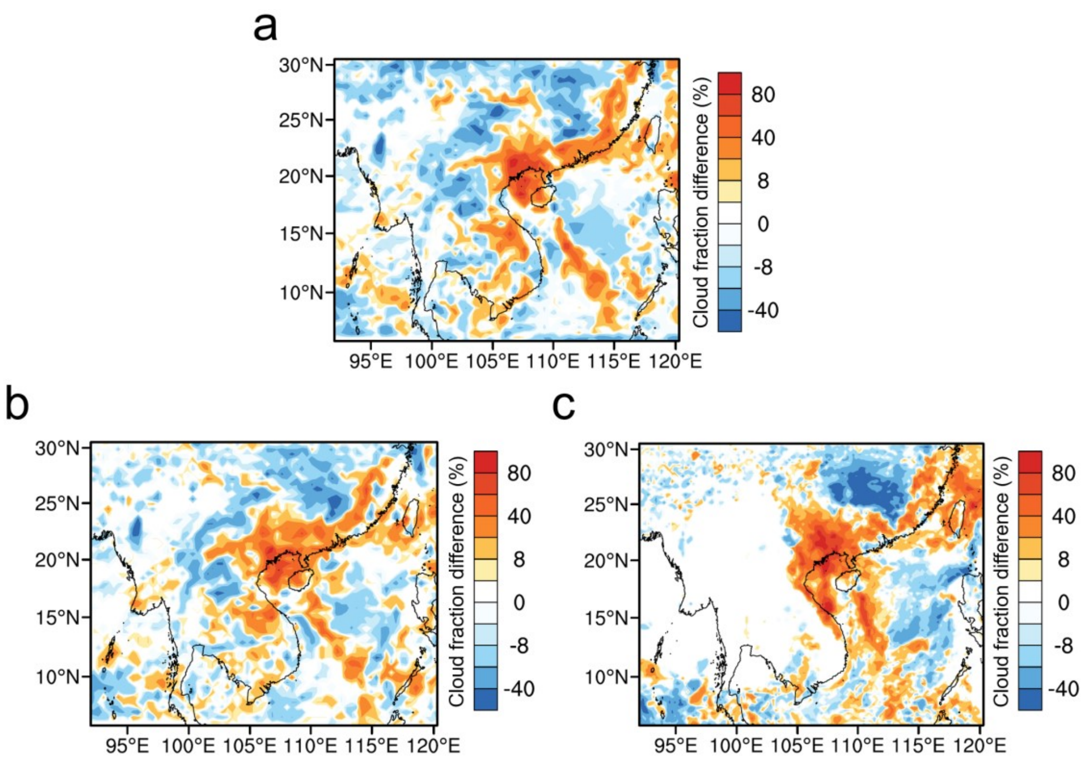

Supplementary Fig. 7 | Comparison of model simulations with different microphysical schemes and model resolutions. a, The cloud fraction bias on 13 March 2004 due to biomass burning (BB) smoke's aerosol-radiation-interaction (ARI) effect (EXP_ARI - EXP_exAR). **b,** Same as (**a**) but for simulations also considering the aerosol-cloud-interaction (ACI) effect. **c,** Same with (**b**) but for a high-resolution simulation. Note: The high-resolution simulations in (**c**) were conducted for nested domains with spatial resolutions of 27 km and 9 km. Note: The model results with different microphysical schemes and resolutions are similar.

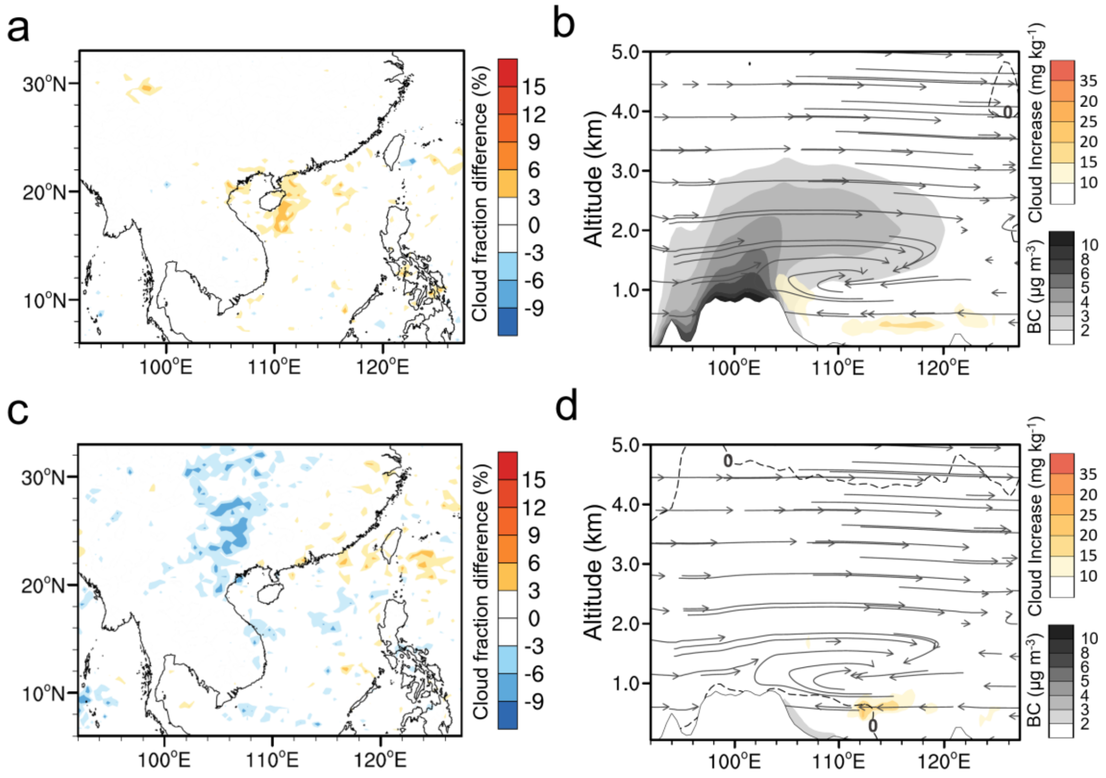

Supplementary Fig. 8 | Impact of different emission sources and interaction processes on cloud fraction in Southeastern Asia. a, Monthly averaged difference of low-cloud fraction (EXP_FAC - EXP_AAC) contributed by aerosol-cloud interaction from biomass burning in Southeast Asia in March 2004. **b,** Vertical distribution of black carbon (BC) and cloud enhancement along the coastal region (17°N – 23°N) corresponding to (**a**). **c,** Same as (**a**) but for aerosol-radiation interaction from fossil fuel combustion sources (EXP_AAR – EXP_exAR). **d,** same as (**b**) but corresponding to (**c**).

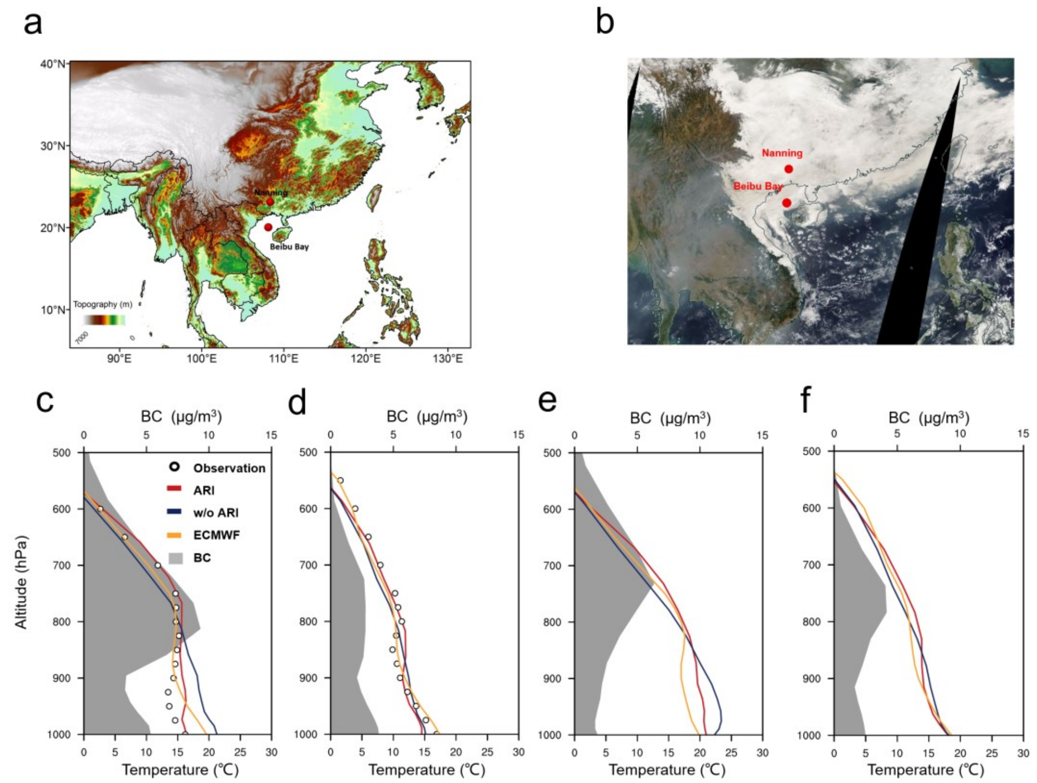

Supplementary Fig. 9 | Comparison of modeling and observed air temperature and clouds at ground stations Nanning and Beibu Bay in March 2004. a, Map showing the topography and location of Nanning and Beibu Bay. **b,** Same as **(a)** but showing the true-color-corrected reflectance images of 13 March 2004 obtained from the NASA Worldview application (https://worldview.earthdata.nasa.gov). **c-d**,Vertical distribution of black carbon (BC) concentration and air temperature from radiosonde measurements and different modeling scenarios for the 8 highest and 8 lowest aerosol-PBL interaction days at Nanning. The temperature profiles are at 20:00 local time, and the BC profiles represent the BC concentration in daytime. **e-f,** Same as (**c**) and (**d**) but over the ocean in Beibu Bay.

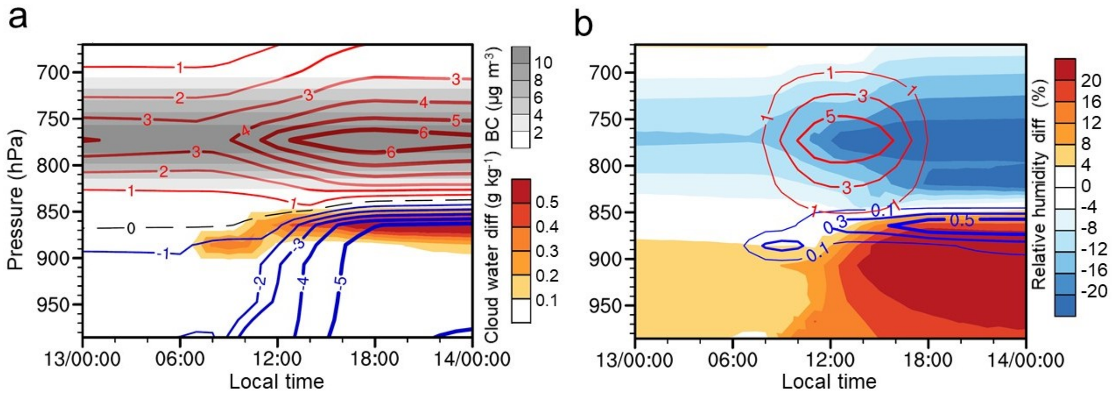

Supplementary Fig. 10 | The role of aerosol-cloud-PBL interactions demonstrated by 1-D WRF-Chem simulations for 13 March 2004 at Wuzhou. a, Changes in air temperature and clouds due to aerosol-cloud-boundary-layer interaction. The red lines and blue lines indicate increase and decrease of air temperature (unit: K). Shaded gray and color contours show black carbon (BC) concentration and change in cloud water. **b,** Changes in relative humidity and short-wave heating due to the influence of aerosols and clouds. The blue contour lines show the increased cloud water (unit: g kg^{-1}) and the red lines show the enhanced short-wave heating rate of aerosols by the aerosol-cloud-boundary-layer interactions (unit: K day^{-1}). Note: The changes in temperature, cloud water and relative humidity are calculated from the difference between the experiments CEXP_AR&CR and CEXP_exAR. The enhanced short-wave heating rate is calculated from the difference between the experiments CEXP_AR&CR and CEXP_AR&exCR.

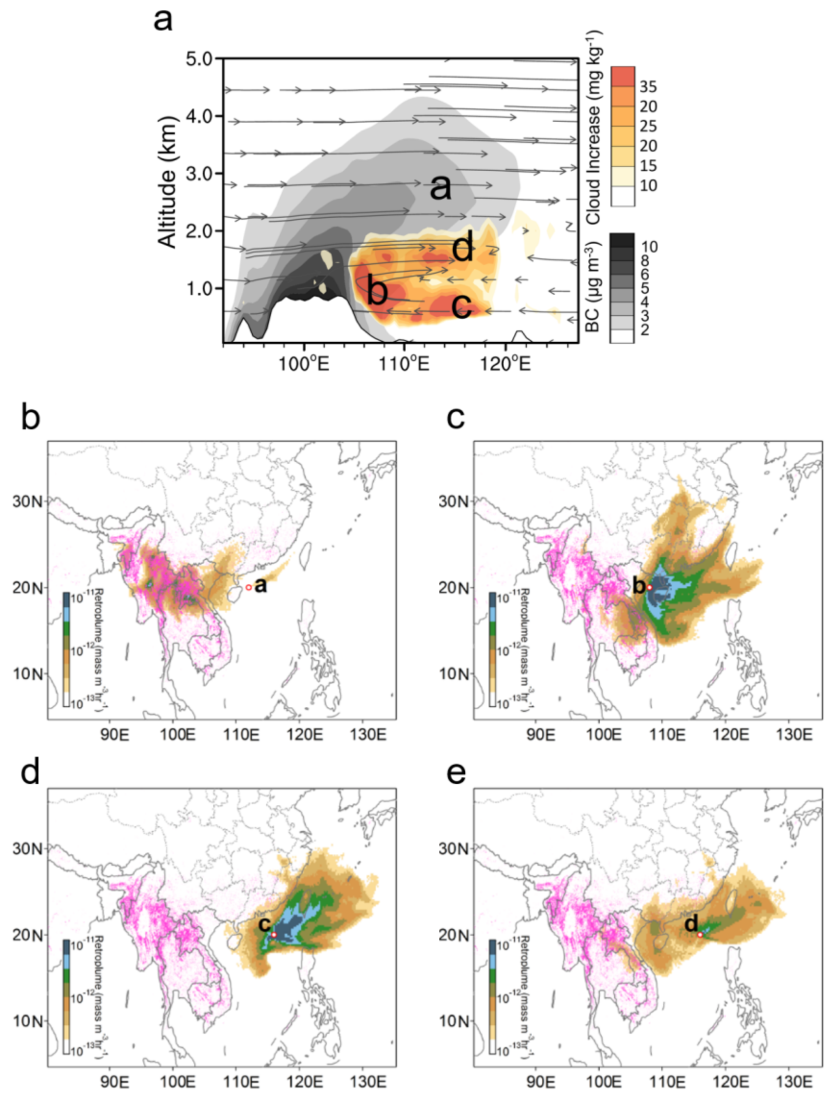

Supplementary Fig. 11 | Air mass origins for smoke and cloud along the BB plumes calculated by a Lagrangian dispersion model. a, averaged vertical cross-section of cloud difference and BC along the coastal line (17°N – 23°N) for the experiments EXP_ARI and EXP_exAR in March 2004 in Asia. **b-e**, Lagrangian dispersion modeling results shows air mass source regions for Points a, b, c, and d. Note: The Lagrangian dispersion modeling was based on the Hybrid Single-Particle Lagrangian Integrated Trajectory (HYSPLIT)[66], following the method described in Ding et al.[67] WRF-Chem simulated meteorological data was used to run the model.

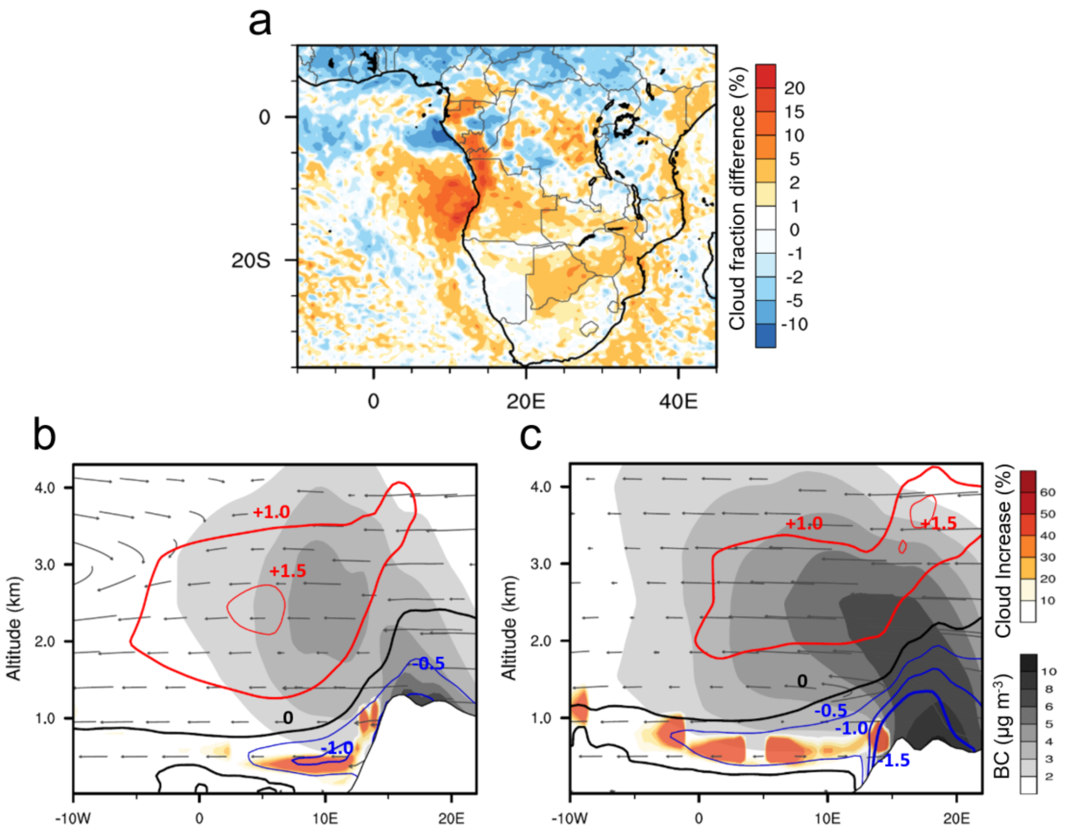

Supplementary Fig. 12 | Biomass burning induced aerosol, clouds, and air temperature changes in central Africa. a, Monthly averaged difference of low cloud fraction for WRF-Chem simulations with fires on/off in Africa during August 2010. **b, c,** Averaged vertical cross-section of black carbon (BC) plumes, relative increase of cloud water and air temperature change (red contours for heating and blue contours for dimming, unit: K) by aerosols aerosol-radiation-interaction (ARI) effect along the belts of smoke-induced low cloud enhancement between latitudes of 8°S –18°S and 4°S – 8°S in Africa during August 2010. Note: Results are from the difference between the experiments EXP_ARI_AA and EXP_exAR_AA (Supplementary Table 3) and BC concentrations are from EXP_ARI_AA.

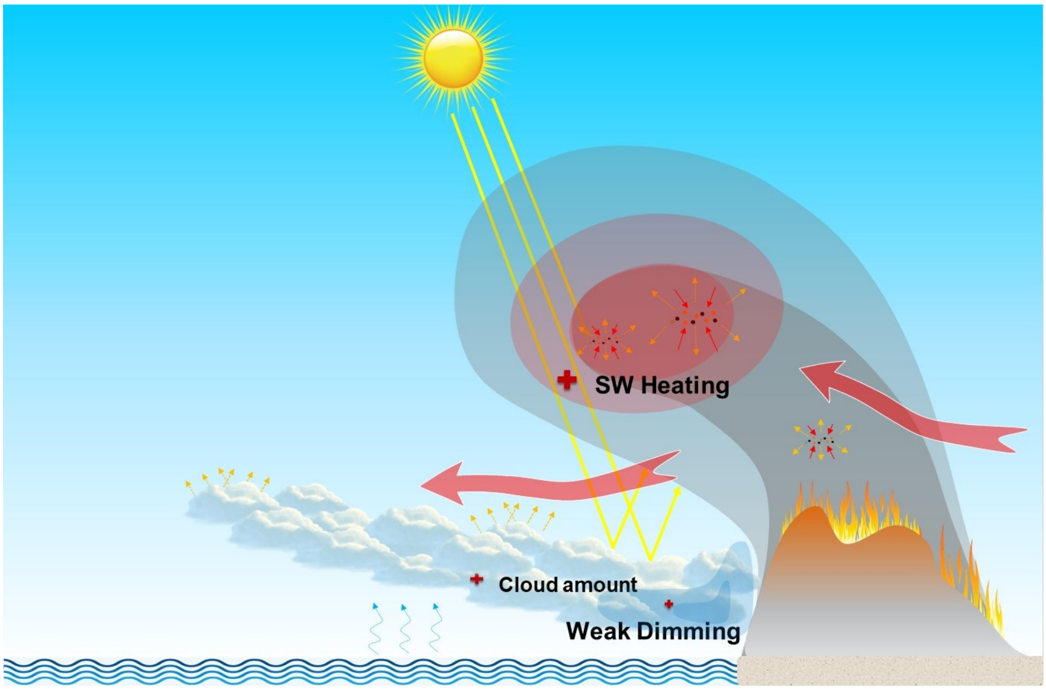

Supplementary Fig. 13 | Schematic figure showing the mechanism of smoke - low cloud interaction in the Atlantic-Africa region. The gray shading indicates the biomass burning plume. The red color along the plume shows shortwave heating by absorbing aerosols like black carbon. The blue shading in the lee side of the plateau indicates the dimming caused by aerosol-cloud-boundary-layer interaction. SW means shortwave.

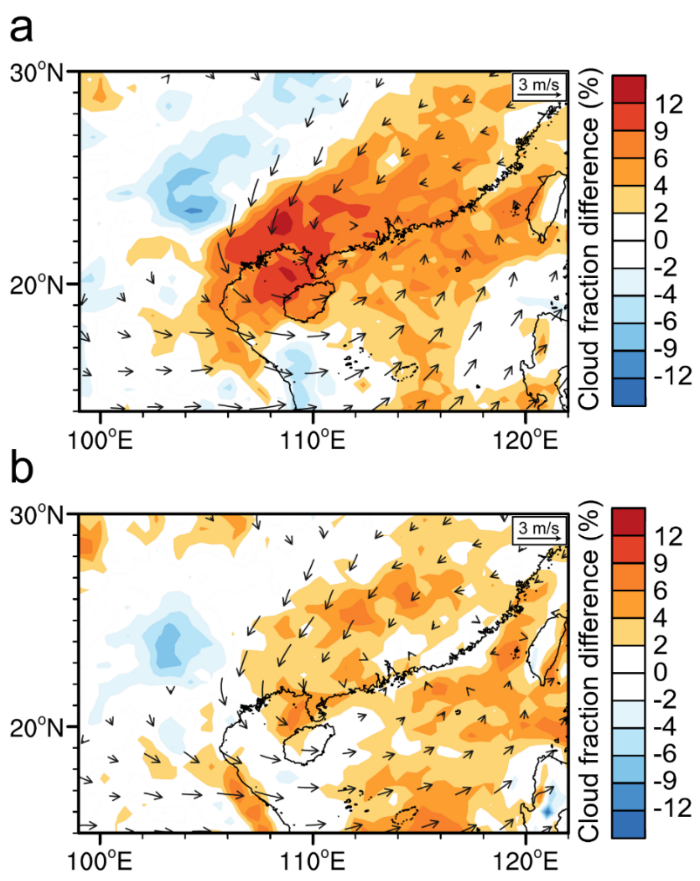

Supplementary Fig. 14 | Increased cloud cover due to synergetic feedback and adjusted monsoon circulation alone in high aerosol optical depth (AOD) years (2004, 2007, 2010 and 2014). a, Increased cloud covers and changed wind fields at the altitude of 1 km due to the aerosol-radiation interaction (ARI) effect of biomass burning (BB) smoke aerosol (EXP_ARI - EXP_exAR). **b,** Increased cloud covers due to the adjusted monsoon circulation (shown as vectors) alone (EXP_ARIwind_exARITemp_ndg-EXP_exAR). Note: EXP_ARI - with aerosol-radiation interaction, EXP_exAR - without aerosol-radiation interaction, EXP_ARIwind_exARITemp_ndg - with wind nudged to that of the simulation with ARI (i.e., the adjusted monsoon circulation) and air temperature nudged to that without ARI effect (i.e., no influence from aerosol-cloud-radiation interaction).

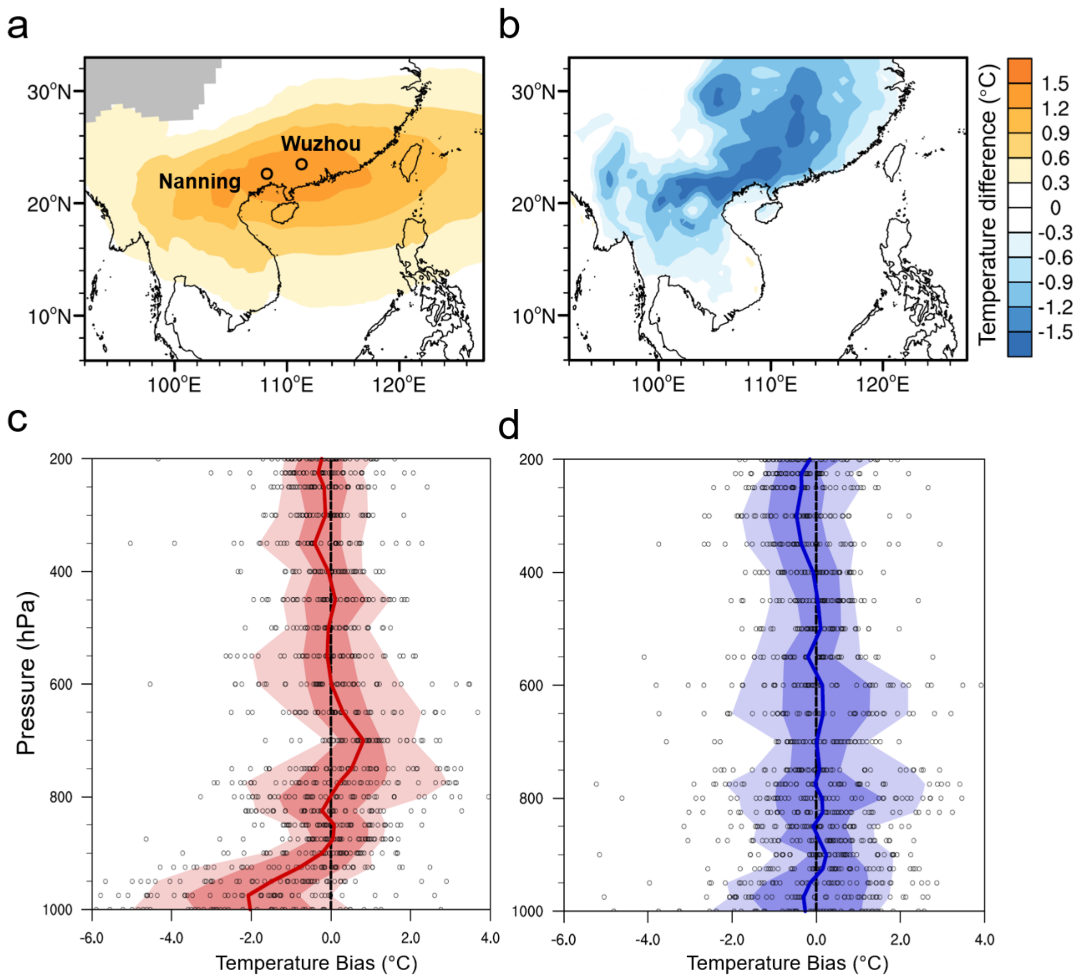

Supplementary Fig. 15 | Comparison of vertical profiles of observation minus reanalysis (OMR) air temperature bias with/without influence from biomass burning. **a-b**, Averaged temperature changed at an altitude of 3 km (about 700-hPa) and on the surface due to the radiation effect of biomass burning aerosols in high aerosol optical depth (AOD) years (2004, 2007, 2010 and 2014). **c**, Vertical profile of OMR air temperature for days with air masses at the altitude of 3 km influenced by biomass burning at Nanning and Wuzhou in Southwest China. **d,** Same as (**c**) but for air masses less influenced by biomass burning. Solid lines and deep and light shadows represent average, 25–75th percentile and 10–90th percentile, respectively. Note: 72-hr back trajectories at 3 km altitude, calculated using HYSPLIT, are used to classify the influence from biomass burning in Southeast Asia. The 50 days with highest/lowest AOD are selected in the statistics here. The locations of Nanning and Wuzhou are given in **(a)**.

Supplementary Table 1 | Averaged carbon emission in four regions with intensive biomass burning activities during 2000-2015.

Domain[a]	Period	Total Carbon Emission (Tg C)
Domain1 –This study	Mar.-Apr.	62
Domain2 –Center Africa	Nov.-Dec.	184
Domain3 –South Africa	Jul.-Aug.	314
Domain4 –South America	Aug.-Sep.	169

[a] Domain 1-4 are marked in Supplementary Fig. 2a.

Supplementary Table 2 | Model configuration options and settings

**Domain setting**		
**Region**	**Asia**	**Africa**
Horizontal grid	90 × 170	140 × 210
Grid spacing	50 km × 50 km	50 km × 50 km
Vertical layers	30 eta levels	30 eta levels
Center point	105 ˚E, 25 ˚N	13 ˚E, 15 ˚S
Map projection	Lambert	Mercator
**Paramerization configuration**		
Long-wave radiation	RRTMG	
Short-wave radiation	RRTMG	
Cumulus parameterization	Grell–Deveny	
Land-surface	Noah	
PBL	MYJ	
Microphysics	Lin et al.	
Photolysis	Fast-J	
Gas chemistry	CBMZ	
Aerosol chemistry	MOSAIC	
**Emission inventories**		
Anthropogenic activities	MIX Asian emission inventory	
Biogenic emissions	online caculated by MEGAN	
Biomass burning emission	caculated by 3BEM using burned area and thermal anomalies dataset	

Supplementary Table 3 | WRF-Chem parallel numerical experiment designs

Experiments		Emissions		Aerosols' radiative effect		Aerosol cloud interaction
		Fossil fuel combustion	Biomass burning	Scattering	Absorption	
ARI EXP	EXP_exAR	√	√	×	×	×
	EXP_ARI	√	√	√	√	×
	EXP_AAR	√	×	√	√	×
	EXP_SAR	√	√	√	×	×
ACI EXP	EXP_FAC	√	√	×	×	√
	EXP_AAC	√	×	×	×	√
Africa-Atlantic ACI EXP	EXP_exAR_AA	×	√	×	×	×
	EXP_ARI_AA	×	√	√	√	×

Supplementary Table 4 | Parallel numerical experiments for WRF-Chem single column simulation

Experiment	Aerosols impact on radiation	Clouds impact on radiation
CEXP_exAR	×	√
CEXP_AR&exCR	√	×
CEXP_AR&CR	√	√
